# Anti-tumor Necrosis Factor Therapy in Inflammatory Bowel Disease: Effectiveness and Challenges in a Moroccan Tertiary Care Center

**DOI:** 10.7759/cureus.112958

**Published:** 2026-07-19

**Authors:** Fatimaezzahra Lairani, Jihane Rizkou, Hala Aouroud, Oussama Nacir, Adil Ait Errami, Sofia Oubaha, Zouhour Samlani, Khadija Krati

**Affiliations:** 1 Gastroenterology and Hepatology, Mohammed VI University Hospital Center, Marrakech, MAR

**Keywords:** anti-tumor necrosis factor therapy, biologic therapy, crohn's disease, inflammatory bowel disease, loss of response, morocco, primary non-response, real-life study, ulcerative colitis

## Abstract

Background: Anti-tumor necrosis factor alpha (anti-TNF-α) agents have transformed the management of moderate-to-severe inflammatory bowel disease (IBD). However, primary non-response, secondary loss of response, and access-related barriers remain important challenges, especially in resource-limited settings.

Aim: The aim of this study was to evaluate the real-life effectiveness of anti-TNF-α therapy in patients with IBD followed in a Moroccan tertiary gastroenterology department and to describe clinical and access-related challenges associated with treatment outcomes in a resource-limited setting.

Methods: We conducted a retrospective single-center study in the Department of Gastroenterology and Hepatology at Mohammed VI University Hospital, Marrakech, from September 2019 to March 2024. Patients with Crohn’s disease (CD) or ulcerative colitis (UC) who were treated with anti-TNF-α therapy and followed in the department were included. Demographic, clinical, therapeutic, and outcome data were collected from medical records. The analysis was primarily descriptive, and results were expressed as numbers and percentages.

Results: Seventy-nine patients were included. The mean age was 36 years, with a range of 11 to 73 years. Of the 79 patients, 43 (54%) were men and 36 (46%) were women. CD was present in 60 patients (76%) and UC in 19 patients (24%). Among patients with CD, ileocolonic involvement was observed in 31 of 60 patients (51.6%), a stenosing phenotype in 30 of 60 patients (50%), and perianal lesions in 34 of 60 patients (56.6%). Among patients with UC, pancolitis was present in 10 of 19 patients (52.6%). Infliximab was used in 56 of 79 patients (71%), adalimumab in 19 of 79 patients (24%), and golimumab in four of 79 patients (5%). Combination therapy with azathioprine was used in 45 of 79 patients (57%). After a mean follow-up of 30 months, primary non-response occurred in 15 of 79 patients (19%), and secondary loss of response occurred in 18 of 79 patients (22.8%). Management strategies included optimization, intra-class switching, ustekinumab, and surgery in selected cases. Six of 79 patients (7.6%) underwent treatment de-escalation. Treatment was discontinued because of financial constraints in 18 of 79 patients (22.8%), and 6 of 79 patients (7.6%) were lost to follow-up. Perianal lesions, colonic involvement, previous intestinal resection greater than 50 cm, and active smoking were observed among patients with treatment failure or loss of response and were interpreted descriptively.

Conclusion: Anti-TNF-α therapy showed real-life effectiveness in this tertiary-center cohort despite a high proportion of severe IBD phenotypes. Primary non-response and secondary loss of response remain frequent challenges. Severe disease features and access-related barriers may influence long-term outcomes. Close monitoring, individualized management, reactive therapeutic drug monitoring when available, and improved access to biologics are essential to optimize care in resource-limited settings.

## Introduction

Inflammatory bowel disease (IBD), including Crohn’s disease (CD) and ulcerative colitis (UC), is a chronic, relapsing inflammatory disorder of the gastrointestinal tract. Its course may be marked by progressive bowel damage, disability, hospitalization, surgery, and impaired quality of life [1,2].

Over the last two decades, the management of IBD has undergone a major diagnostic and therapeutic revolution. Treatment objectives have moved beyond simple symptom control toward a treat-to-target strategy that aims for sustained clinical remission, biomarker normalization, mucosal healing, prevention of disability, and improved quality of life [3].

Biologic therapies, particularly anti-tumor necrosis factor alpha (anti-TNF-α) agents, remain key treatments for moderate-to-severe IBD. Infliximab and adalimumab are widely used in CD and UC, especially in steroid-dependent, steroid-refractory, fistulizing, or immunosuppressant-refractory disease [4,5]. Other therapeutic classes, including anti-integrins and anti-interleukin agents, as well as small molecules such as Janus kinase inhibitors, have expanded the therapeutic armamentarium. However, access to these alternatives may remain limited in resource-constrained settings [5,6].

Despite their proven efficacy, anti-TNF-α agents are not universally effective. Primary non-response and secondary loss of response remain major obstacles and require close monitoring, dose optimization, intra-class switching, changing to another mechanism of action, or surgical intervention when appropriate [7-9]. Pharmacokinetic variability, immunogenicity, disease phenotype, inflammatory burden, and treatment interruptions may all contribute to therapeutic failure [7-10].

In countries with limited resources, real-life evaluation of anti-TNF-α effectiveness is particularly important. Treatment cost, drug availability, incomplete access to therapeutic drug monitoring (TDM), and delayed access to newer biologics may influence both treatment persistence and clinical outcomes [11]. This study aimed to evaluate the real-life effectiveness of anti-TNF-α therapy in patients with IBD followed in a Moroccan tertiary-care center and to describe clinical and access-related challenges associated with treatment outcomes in a resource-limited setting.

## Materials and methods

Study design and setting

This was a retrospective, analytical, single-center study conducted in the Department of Gastroenterology and Hepatology at Mohammed VI University Hospital, Marrakech, Morocco. The study period extended from September 2019 to March 2024.

Study population

We included all patients followed for IBD, either CD or UC, who received anti-tumor necrosis factor alpha (anti-TNF-α) therapy during the study period. The diagnosis of IBD was based on clinical, endoscopic, histological, biological, and/or radiological criteria.

Inclusion and exclusion criteria

Patients were eligible if they had a confirmed diagnosis of CD or UC, had received at least one anti-TNF-α agent, and had available clinical and therapeutic follow-up data. Patients were excluded when the diagnosis of IBD was uncertain, medical records were incomplete, or follow-up data were insufficient to evaluate therapeutic response.

Data collection

Data were collected from medical records, outpatient follow-up files, hospitalization records, endoscopy reports, biological results, and therapeutic follow-up documents. The following variables were collected: age, sex, type of IBD, disease location and phenotype, perianal lesions, extraintestinal manifestations, indications for anti-TNF-α therapy, prescribed anti-TNF-α agent, combination therapy with azathioprine, allergic reactions, primary non-response, secondary loss of response, therapeutic strategies after failure, treatment de-escalation, discontinuation because of financial constraints, and loss to follow-up.

Definitions

Primary non-response was defined as the absence of meaningful clinical improvement after induction therapy with anti-TNF-alpha. Secondary loss of response was defined as recurrence of disease activity or clinical worsening after an initial response to anti-TNF-α therapy. Treatment optimization included dose escalation or shortening of the administration interval. Switch referred to changing from one anti-TNF-α agent to another anti-TNF-α agent. Swap referred to changing to another therapeutic class, mainly ustekinumab, in this cohort. Treatment discontinuation due to financial constraints referred to anti-TNF-α interruption because of lack of means or access-related barriers, particularly during the RAMED (Régime d'Assistance Médicale) period and before broader AMO coverage and biologic assistance programs. Loss to follow-up was defined as an interruption of regular follow-up without a documented planned transfer of care.

Statistical analysis

Qualitative variables were expressed as numbers and percentages. Quantitative variables were expressed as means and ranges when available. Because of the retrospective design and the use of aggregated data extracted from medical records, the analysis was primarily descriptive. Variables potentially associated with treatment failure or loss of response were explored descriptively by reporting the corresponding counts and percentages. No multivariable analysis was performed because of the limited sample size. Statistical analyses were interpreted cautiously and were considered exploratory.

## Results

Baseline characteristics

A total of 79 patients treated with anti-TNF-α therapy were included. The mean age was 36 years, with a range of 11 to 73 years. There were 43 men (54%) and 36 women (46%), corresponding to a male-to-female sex ratio of 1.19. Crohn’s disease was the most frequent diagnosis, observed in 60 patients (76%), while ulcerative colitis was present in 19 patients (24%). The baseline characteristics of the study population are summarized in Table 1.

**Table 1:** Baseline characteristics of the study population M/F: male-to-female ratio.

Variable	Result
Total number of patients	79
Mean age	36 years (range: 11–73)
Men	43 (54%)
Women	36 (46%)
Sex ratio M/F	1.19
Crohn’s disease	60 (76%)
Ulcerative colitis	19 (24%)

Disease phenotype and location

Among patients with Crohn’s disease, severe phenotypes were highly represented. Ileocolonic location was observed in 31 of 60 patients (51.6%), stenosing phenotype in 30 of 60 patients (50%), and perianal lesions in 34 of 60 patients (56.6%). Among patients with ulcerative colitis, pancolitis was present in 10 of 19 patients (52.6%). Extraintestinal manifestations were reported in 25 of 79 patients (31.6%) and were mainly articular. Disease phenotype and location are shown in Table 2.

**Table 2:** Disease phenotype and location CD: Crohn’s disease; UC: ulcerative colitis.

Disease feature	Result
Ileocolonic location among CD patients	31/60 (51.6%)
Stenosing phenotype among CD patients	30/60 (50%)
Perianal lesions among CD patients	34/60 (56.6%)
Pancolitis among UC patients	10/19 (52.6%)
Extraintestinal manifestations	25 patients, mainly articular

Indications for anti-TNF-α therapy

The main indication for anti-TNF-α therapy was perianal disease, reported in 34 of 79 patients (43%). Other indications included immunosuppressant failure in 17 of 79 patients (21.5%), immunosuppressant intolerance in 14 of 79 patients (17.7%), steroid resistance in 12 of 79 patients (15.2%), steroid dependence in 12 of 79 patients (15.2%), progressive fistulizing Crohn’s disease in 11 of 79 patients (13.9%), postoperative recurrence in four of 79 patients (5.1%), and upper gastrointestinal involvement in three of 79 patients (3.8%). The indications for anti-TNF-α therapy are presented in Table 3.

**Table 3:** Indications for anti-TNF-α therapy Anti-TNF-α: anti-tumor necrosis factor-alpha.

Indication	Number of patients
Perianal lesions	34
Immunosuppressant failure	17
Immunosuppressant intolerance	14
Steroid resistance	12
Steroid dependence	12
Progressive fistulizing Crohn’s disease	11
Postoperative recurrence	4
Upper gastrointestinal involvement	3

Anti-TNF-α agents and combination therapy

Infliximab was the most frequently prescribed anti-TNF-α agent, used in 56 of 79 patients (71%), followed by adalimumab in 19 of 79 patients (24%) and golimumab in four of 79 patients (5%). Golimumab was included because it was prescribed at the beginning of the study period. Combination therapy with azathioprine was used in 45 of 79 patients (57%), mainly to optimize treatment response and reduce immunogenicity. Allergic reactions occurred in 6 of 79 patients (7.6%) and required a switch between infliximab and adalimumab. Anti-TNF-α agents and associated treatments are summarized in Table 4.

**Table 4:** Anti-TNF-α agents and associated treatments Anti-TNF-α: anti-tumor necrosis factor-alpha.

Treatment variable	Result
Infliximab	56 (71%)
Adalimumab	19 (24%)
Golimumab	4 (5%)
Combination therapy with azathioprine	45 (57%)
Allergic reaction requiring switch	6 (7.6%)

Therapeutic outcomes

The mean follow-up duration was 30 months. Primary non-response occurred in 15 of 79 patients (19%). Management strategies for primary non-response included optimization in 7 of 15 patients (46.7%), intra-class switch in 2 of 15 patients (13.3%), ustekinumab in 4 of 15 patients (26.7%), and surgery in 2 of 15 patients (13.3%).

Secondary loss of response occurred in 18 of 79 patients (22.8%). Optimization was used in 3 of 18 patients (16.7%), a switch in 8 of 18 patients (44.4%), and ustekinumab in 7 of 18 patients (38.9%). Six of 79 patients (7.6%) underwent treatment de-escalation, reflecting favorable disease evolution in selected cases. Therapeutic outcomes and strategies after treatment failure are shown in Table 5.

**Table 5:** Therapeutic outcomes and strategies after failure

Outcome or strategy	Result
Mean follow-up	30 months
Primary non-response	15 (19%)
Optimization after primary non-response	7
Intra-class switch after primary non-response	2
Ustekinumab after primary non-response	4
Surgery after primary non-response	2
Secondary loss of response	18 (22.8%)
Optimization after secondary loss of response	3
Switch after secondary loss of response	8
Ustekinumab after secondary loss of response	7
Treatment de-escalation	6

Access-related discontinuation and loss to follow-up

Treatment was discontinued because of financial constraints in 18 of 79 patients (22.8%). This mainly concerned the RAMED period and preceded broader AMO coverage and public-sector biologic assistance programs, which subsequently improved access to biologic therapy. Six of 79 patients (7.6%) were lost to follow-up, representing an important limitation inherent to retrospective real-life studies. Access-related outcomes are presented in Table 6.

**Table 6:** Access-related outcomes

Variable	Result
Treatment discontinuation due to financial constraints	18 patients
Loss to follow-up	6 patients

Clinical factors observed in patients with treatment failure or loss of response

In this cohort, several clinical factors were observed among patients with treatment failure or loss of response, including perianal lesions in 34 of 60 patients with Crohn’s disease (56.6%), colonic involvement in 31 of 60 patients with Crohn’s disease (51.6%), previous intestinal resection greater than 50 cm in 10 of 79 patients (12.7%), and active smoking in 20 of 79 patients (25.3%). These factors reflect severe or complicated disease profiles and were considered clinically relevant in the interpretation of anti-TNF-α treatment outcomes, as summarized in Table 7.

**Table 7:** Clinical factors observed in patients with treatment failure or loss of response Values are presented as n/N (%). Because individual-level cross-tabulated data were not available for all variables, these findings were interpreted descriptively.

Factor	Number of patients, n (%)
Perianal lesions	34/60 (56.6%)
Colonic involvement	31/60 (51.6%)
Previous intestinal resection > 50 cm	10/79 (12.7%)
Active smoking	20/79 (25.3%)

## Discussion

This study provides real-life data on the effectiveness and challenges of anti-TNF-α therapy in patients with IBD followed in a Moroccan tertiary center. The cohort was characterized by a high proportion of CD, severe phenotypes, perianal disease, stenosing behavior, and ileocolonic involvement. This reflects the recruitment pattern of a tertiary referral center, where patients frequently present with complicated or treatment-refractory disease [1,4].

Anti-TNF-α agents have revolutionized IBD care since the late 1990s. Infliximab and adalimumab are recommended for the induction and maintenance of remission in moderate-to-severe CD and UC, and infliximab has a specific historical role in fistulizing perianal CD [4-6,12]. In our cohort, infliximab was the most commonly used agent, consistent with its frequent use in severe and fistulizing disease. Adalimumab represented the second most frequently used anti-TNF-α agent, while golimumab was included because it was prescribed earlier during the study period.

The rate of primary non-response in our cohort was 19%, and the rate of secondary loss of response was 22.8%. These rates are within the range reported in the literature, where primary non-response to anti-TNF-α therapy may occur in approximately 10% to 40% of patients and loss of response may affect a substantial proportion during maintenance therapy [7,8,13]. This concordance suggests that, despite a severe disease profile, the therapeutic outcomes observed in our real-life cohort are broadly comparable with international data.

Anti-TNF-α failure is multifactorial. Pharmacokinetic variability, low drug concentrations, anti-drug antibodies, immunogenicity, severe inflammatory burden, and mechanistic failure may all contribute to primary non-response or secondary loss of response [7-10,14]. The PANTS (personalized anti-TNF therapy in Crohn's disease study) study showed that immunogenicity and suboptimal drug exposure are important determinants of anti-TNF-α failure in CD [10]. More recent evidence has confirmed the importance of therapeutic drug monitoring and individualized optimization strategies in patients with an inadequate response [9,14].

In our study, perianal lesions were observed among patients with treatment failure or loss of response. This finding is clinically relevant because perianal CD is a severe phenotype requiring multidisciplinary management, including gastroenterologists, colorectal surgeons, and radiologists [12,15]. Sustained fistula control often requires a combined strategy that includes drainage, seton placement, antibiotics, immunosuppressants, anti-TNF-α therapy, and, sometimes, surgical intervention [12,15].

Colonic involvement, previous intestinal resection greater than 50 cm, and active smoking were also observed among patients with treatment failure or loss of response. These factors may reflect more severe, extensive, or complicated disease profiles rather than statistically confirmed predictors, given the retrospective design and the absence of complete individual-level cross-tabulated data. Active smoking remains a well-known negative prognostic factor in CD and has been associated with disease progression, postoperative recurrence, and poorer therapeutic outcomes [16,17]. These observations should be interpreted as exploratory and descriptive rather than as evidence of causal relationships or independent predictors of treatment failure, given the retrospective single-center design, limited sample size, and absence of multivariable statistical analysis.

The therapeutic strategies used in our department for anti-TNF-α failure were consistent with international algorithms. These included optimization, intra-class switching, and switching to ustekinumab, with surgery reserved for selected cases. In modern practice, the choice between optimization, switching, or swapping should ideally be guided by reactive therapeutic drug monitoring, assessment of inflammatory activity, and endoscopic or imaging reassessment [9,14,18]. However, in resource-limited settings, systematic access to therapeutic drug monitoring and newer biologics may be restricted, influencing therapeutic decisions.

An important finding of this study is the role of access-related barriers. Eighteen patients discontinued therapy because of financial constraints. This mainly occurred during the Medical Assistance Scheme (Régime d'Assistance Médicale, RAMED) period and before the broader implementation of Mandatory Health Insurance (Assurance Maladie Obligatoire, AMO) coverage and biologic assistance programs in the public sector. This point is crucial in real-life studies because treatment effectiveness depends not only on pharmacological efficacy but also on treatment continuity, availability, affordability, and adherence to scheduled administration [11].

Our findings support the need for structured IBD care pathways. Because IBD phenotypes, patient profiles, and therapeutic options are highly heterogeneous, management cannot be systematic or uniform. Instead, individualized treatment decisions, close monitoring, early recognition of failure, and reactive therapeutic drug monitoring when available are necessary to maximize anti-TNF-α benefit and improve long-term outcomes [3,9,18].

This study has several limitations. First, it was retrospective and based on medical records, which may result in missing data. Second, the sample size was limited, reducing the power of subgroup analyses. Third, therapeutic drug monitoring and anti-drug antibody testing were not systematically available. Fourth, endoscopic healing and biomarker normalization could not be assessed in all patients. Finally, the single-center design may limit generalizability. Despite these limitations, this work provides clinically relevant real-world data on anti-TNF-α effectiveness and challenges in a Moroccan tertiary-care context. In addition, the study primarily used descriptive analysis and did not include multivariable modeling; therefore, the clinical factors observed among patients with treatment failure or loss of response should be interpreted as exploratory observations rather than independent predictors or causal associations. Standardized disease activity indices, such as the Crohn’s Disease Activity Index or Mayo score, objective inflammatory biomarkers, therapeutic drug monitoring, and predefined response criteria were not consistently available in all medical records, which may affect reproducibility and comparability with other studies. Finally, selection bias and missing data inherent to retrospective chart reviews may have influenced the findings.

## Conclusions

Anti-TNF-α therapy represents a major therapeutic advance in the management of moderate-to-severe IBD. In this real-life tertiary-center cohort, anti-TNF-α agents showed effectiveness despite a high proportion of severe phenotypes, particularly perianal and ileocolonic Crohn’s disease. Nevertheless, primary non-response and secondary loss of response remain frequent challenges in routine practice.

Perianal lesions, colonic involvement, previous intestinal resection greater than 50 cm, and active smoking were observed among patients with therapeutic failure and may reflect more severe or complicated disease profiles. These observations should be interpreted as exploratory and descriptive rather than as independent predictors, given the retrospective single-center design and descriptive analysis. These findings reinforce the need for individualized treatment strategies, close surveillance, early identification of non-response, and reactive therapeutic drug monitoring whenever available. Improving access to biologic therapy and ensuring continuity of care remain essential to optimize long-term outcomes in resource-limited settings.
